# Effect of different disinfection protocols in bacterial viability of an intraradicular biofilm formed *in situ*

**DOI:** 10.1590/0103-6440202305244

**Published:** 2023-07-17

**Authors:** Felipe Barros Matoso, Francisco Montagner, Alexander Pompermayer Jardine, Ramiro Martins Quintana, Fabiana Soares Grecca, Patricia Maria Poli Kopper

**Affiliations:** 1 Graduate Program in Dentistry, Federal University of Rio Grande do Sul (UFRGS), Porto Alegre, RS, Brazil.

**Keywords:** Biofilms, Endodontics, Root Canal Therapy, Root Canal Preparation, Photodynamic Therapy

## Abstract

The present study aimed to evaluate bacterial viability after the use of different disinfection protocols in root canals infected with a multispecies biofilm (MB) formed *in situ*. Palatal roots with a single canal were obtained from extracted maxillary molars and sterilized before being inserted into the mouth. The roots were contaminated with a MB in an intraoral appliance worn by ten volunteers. All volunteers wore six roots simultaneously in two intraoral devices for 21 days. One root from each volunteer was assigned to each group (n=10): PUI - passive ultrasonic irrigation; EC - Easy Clean; XPF - XP-endo Finisher; aPDT - antimicrobial photodynamic therapy; CI - conventional irrigation; and NC - negative control. The samples were evaluated under confocal laser scanning microscopy. The percentage of viable cells (VC) was calculated over the total percentage of MB biovolume. Data were statistically analyzed (α=5%). The cell viability in the entire root canal or for each third was compared between groups (Kruskal-Wallis test, Dunn post-hoc test) and for the same group (Friedman test, Dunn post-hoc test). Disinfection protocols were not significantly different from each other (P>.05). Samples in EC, PUI, and aPDT had lower cell viability than in NC (P<.05). In the coronal third of samples in the EC, XPF, PUI and aPDT, the percentage of VC biovolume was lower than in the NC (P<.05). The percentage of VC in EC samples was lower in the coronal and middle thirds than in the apical third (P<.05). EC, PUI and aPDT had significant effects on cell viability in intraradicular multispecies biofilm formed in situ when compared with untreated samples.

## Resumo

O presente estudo teve como objetivo avaliar a viabilidade bacteriana após o uso de diferentes protocolos de desinfecção em canais radiculares infectados com um biofilme multiespécies (MB) formado *in situ*. Raízes palatinas com canal único foram obtidas de molares superiores extraídos e esterilizadas antes de serem inseridas na boca. As raízes foram contaminadas com MB em um aparelho intraoral usado por dez voluntários. Todos os voluntários usaram seis raízes simultaneamente em dois dispositivos intrabucais por 21 dias. Uma raiz de cada voluntário foi atribuída a cada grupo (n=10): PUI - irrigação ultrassônica passiva; EC - Easy clean; XPF - XP-endo Finisher; aPDT - terapia fotodinâmica antimicrobiana; IC - irrigação convencional; e, NC - controle negativo. As amostras foram avaliadas em microscopia confocal de varredura a laser. A porcentagem de células viáveis (VC) foi calculada sobre a porcentagem total do biovolume de MB. Os dados foram analisados estatisticamente (α=5%). A viabilidade celular em todo o canal radicular ou em cada terço foi comparada entre os grupos (teste de Kruskal-Wallis, teste post-hoc de Dunn) e no mesmo grupo (teste de Friedman, teste post-hoc de Dunn). Os protocolos de desinfecção não foram significativamente diferentes entre si (P>0,05). Amostras dos grupos EC, PUI e aPDT apresentaram menor viabilidade celular do as do NC (P<0,05). No terço cervical das amostras do EC, XPF, PUI e aPDT, a porcentagem de biovolume de VC foi menor do que no NC (P<0,05). A porcentagem de VC nas amostras do EC foi menor nos terços cervical e médio do que no terço apical (P<0,05). EC, PUI e aPDT tiveram efeitos significativos na viabilidade celular do biofilme multiespécies intrarradicular formado *in situ* quando comparado com amostras não tratadas. Estudos clínicos devem investigar o papel da redução de cargas bacterianas viáveis no sistema de canais radiculares para o sucesso do tratamento endodôntico.

## Introduction

Microorganisms, organized in the root canal system as a biofilm, may remain viable even under adverse conditions for their growth^1^. Biofilms hold nutrients and enable metabolic cooperation between various bacteria of the same or different species^2^. Therefore, bacteria that are resistant to cleaning and shaping may be responsible for endodontic failure ^3^. Recent reports indicated that procedures to enhance infection control in the non-surgical endodontic treatment, such as replacing rubber dams, gloves, files, instruments, and surface barriers during canal filling, may favor microbial reduction and apical healing^4^ ^,^ ^5^. Different complementary disinfection protocols have been investigated as alternative methods to reduce bacterial viability and improve root canal cleaning after preparation. Recently, Tonini et al.^6^ indicated that activation methods of irrigants provide significantly higher biofilm reduction than conventional needle irrigation methods. Passive ultrasonic irrigation (PUI), automated instruments for irrigant agitation, and antimicrobial photodynamic therapy (aPDT) have been recommended as supplementary protocols for canal disinfection, in addition to conventional needle irrigation (CI)^7^ ^,^ ^8^ ^,^ ^9^.

PUI has significantly reduced microbes in the root canal *in vitro* trials^7^ ^,^ ^9^ ^,^ ^10^. However, despite some promising results on infection reduction, a systematic review found no evidence of a significant effect on the improvement of root canal disinfection and, consequently, of periapical healing^11^. XP-endo Finisher (XPF; FKG, La Chaux-de-Fonds, Switzerland) and Easy Clean (EC; Easy, Belo Horizonte, Brazil) are engine-driven files that have been developed for the agitation of irrigants after root canal cleaning and shaping as alternatives to the use of PUI. However, reports of their effect on root canal disinfection are contradictory^7^ ^,^ ^9^ ^,^ ^12^ ^,^ ^13^.

Laser applications have also been studied as adjunctive antimicrobial therapies for root canal disinfection, and findings suggest that they should be used as a supplementary antimicrobial therapy after endodontic debridement^14^ ^,^ ^15^. Antimicrobial photodynamic therapy uses light of a specific wavelength to activate a nontoxic photoactive dye, called a photosensitizer, and generate highly reactive oxygen, which binds to microbial cell membranes and destroys them ^16^.

A considerable number of studies have evaluated different procedures for the disinfection of root canals^7^ ^,^ ^9^ ^,^ ^10^ ^,^ ^12^ ^,^ ^13^ ^,^ ^14^ ^,^ ^15^ ^,^ ^17^ ^,^ ^18^ ^,^ ^19^. However, there is no consensus in the literature about the effect of supplementary disinfection protocols on intraradicular multispecies biofilms under experimental conditions to simulate root canals with pulp necrosis. This study used confocal laser scanning microscopy (CLSM) to analyze the effect of PUI, EC, XPF, aPDT and CI used after cleaning and shaping on the cell viability of a multispecies biofilm formed *in situ*. The null hypothesis was that the different disinfection protocols would not affect the cell viability of intraradicular multispecies biofilms formed *in situ*.

## Material and methods

### Ethical considerations

This study was approved by the Ethics in Research Committee of the Federal University of Rio Grande do Sul, Brazil (CAAE: 01688918.8.0000.5347) and registered on SISGEN (Protocol A09C4B7).

### Sample preparation

Sixty-two extracted maxillary molars with a closed apex and without internal resorption or endodontic treatment were stored in 0.001% NaOCl at 4º C until use. Palatal roots were sectioned close to the cementoenamel junction to prepare standard 17-mm-long samples. Roots were included if a #20 K-file (Sirona Dentsply, York, PA) could be run along the canal and juxtaposed to the canal walls (Figure 1.1).

First, a #10 K file (Sirona Dentsply, York, PA) was used for canal exploration. Working length (WL) was set at 1 mm short of the apical foramen. To standardize canal diameter, the ProDesign Logic 25.01 (Easy, Belo Horizonte, MG, Brazil) file was used for the glide path, and then the ProDesign Logic 25.06 (Easy) shaped the canal. The instruments were used in a rotary movement using a VDW Silver system (VDW GmbH, Munich, Germany) and the DR’s choice program for individual speed and torque settings. Torque was 100 g.cm^2^ at 350 rpm for the 25.01 file and 400 g.cm^2^ at 950 rpm for the 25.06 file. Before and after each instrument was used, the canals were irrigated with 2 mL of distilled water using a disposable plastic syringe (Ultradent Products Inc., South Jordan, UT). An aspiration cannula and a silicone capillary tip (Ultradent Products Inc., South Jordan, UT) were used for aspiration during irrigation.

After initial preparation, the roots were sectioned (Lab-Cut Model 1010, EXTEC, Enfield, CT) into two halves longitudinally. The internal surface of each half was polished using 120-, 280- and 400-grit wet sandpaper strips (Norton, Guarulhos, Brazil). After that, the samples were rinsed in an ultrasonic bath using 20 mL of detergent (Tween® 80, SIGMA, Saint Louis, MO) for three cycles of five minutes each, with detergent replacement after each cycle. Then, a cycle of five minutes with 10 mL of EDTA (Biodinâmica, Ibiporã, Brazil) was performed. The samples were autoclaved for 30 min at 121°C and 1 atm and then individually immersed in Falcon tubes (MyLabor, São Paulo, Brazil) containing 5 mL of distilled water. Two roots selected randomly to control sterility were kept in brain heart infusion (BHI) broth (KASVI, Curitiba, Brazil) for 24 h at 37º C. There was no turbidity in the culture medium after that time (Figure 1.2).

### Intraradicular multispecies biofilm formed *in situ*

Ten volunteers of both sexes aged 21 to 35 years were selected to wear intraoral appliances. They had no caries, gingivitis, periodontal or systemic diseases, were not wearing orthodontic appliances or undergoing tooth bleaching, were not smokers, and did not receive any antimicrobial treatment in the two months before the study or while wearing the appliance.

**Figure 1:**
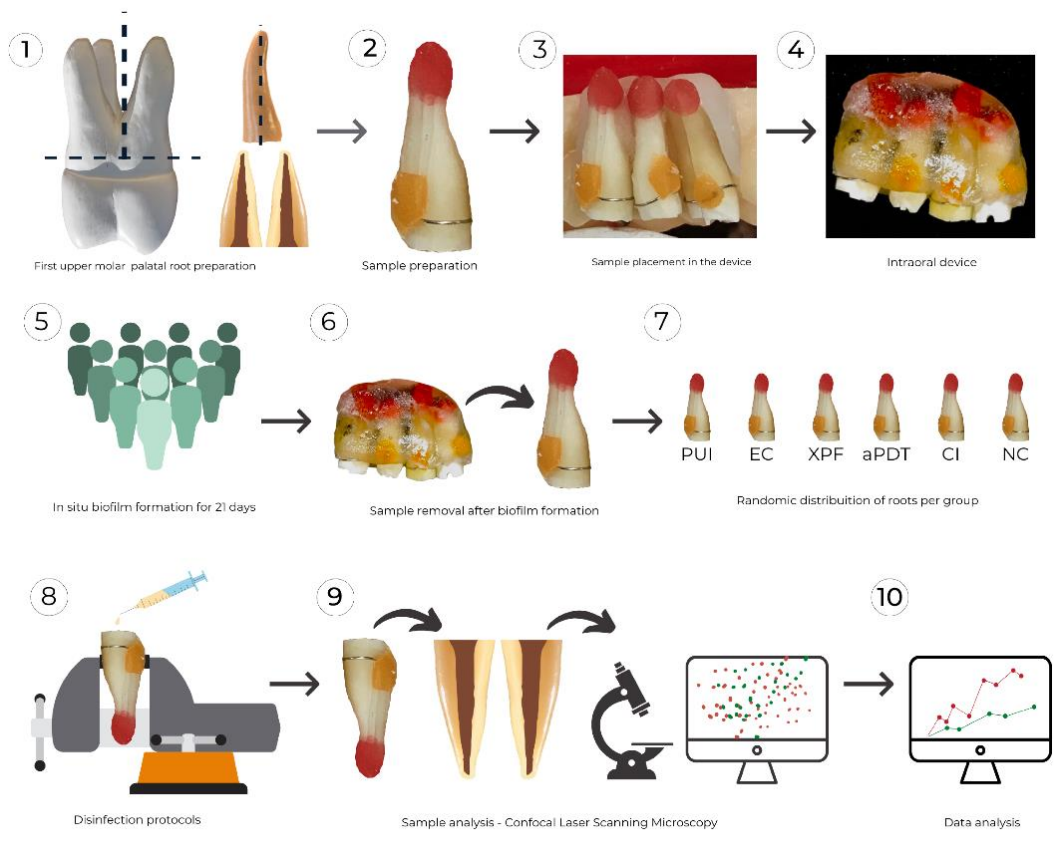
Study design flowchart

The intraoral prosthetic appliances were fabricated using an adaptation of the method described by Barthel et al. 2002^20^. Briefly, impressions of the maxillary arch of the volunteers were taken. Two devices for each volunteer were fabricated using transparent self-curing acrylic resin (Jet, Artigos Odontológicos Clássico, São Paulo, Brazil). They had lateral slots for the placement of the roots. The two halves of the roots were juxtaposed and fastened to the device using a piece of orthodontic wire and utility wax. Six roots (one for each experimental and control group) were assigned to each volunteer, divided into two intraoral devices with three roots each, one for the right and the other for the left side, and kept in the mouth simultaneously. The apical portion of the canal was sealed with wax, the root canal access faced the occlusal plane, and the long axis of the root stayed parallel to the long axis of the volunteer’s teeth. The right and left devices were worn simultaneously by each volunteer for 21 days for biofilm formation and growth ^20^. During this time, they were removed from the mouth and stored in humid gauze only for eating and oral hygiene. Volunteers used a medium bristle toothbrush without dentifrice to clean the resin surfaces of the intraoral device three times a day after the main meals (Figures 1. 3 to 1.5).

Twenty-one days later, immediately after removing the device from the participant's mouth, the roots were extracted from the appliances and randomly divided into 5 experimental groups: PUI, EC, XPF, aPDT, CI and a negative control group (NC) (n=10). The roots were individually stored in Falcon tubes containing reduced transport fluid until the moment of root canal cleaning and shaping (Figures 1.6 and 1.7).

### Experimental design

Shortly after the teeth were removed from the appliance, the root canal of all samples, except of those in the negative control group (no treatment), were prepared by a single trained operator using the ProDesign Logic 40.05 (400 gcm2 and 950 rpm; Easy, Belo Horizonte, Brazil) file to the WL. Before and after the use of the file, the canals were irrigated with 2 mL of 2.5% NaOCl (Asfer, São Caetano do Sul, Brazil) using a syringe (Ultradent, South Jordan, UT) and needle (NaviTip 30; Ultradent, South Jordan, UT) calibrated to 2 mm short of WL, and simultaneously aspirated using a capillary tip (Ultradent).

After that, the protocols for final cleaning were applied (Table 1). In all groups, the total irrigant volume was 6 mL of 2.5% NaOCl at 37ºC, 5 mL of 17% EDTA and 3 mL of distilled water. Irrigation and aspiration were simultaneous. The canals were kept full of NaOCl during the use of the files in the PUI, EC, and XPF groups. In the aPDT group, the canals were irrigated with 6 mL of 2.5% NaOCl, 5 mL of 17% EDTA and 1.5 mL of distilled water. They were then dried and filled with 1 mL of 0.005% methylene blue for 2 minutes. The optical fiber was then inserted into the canal and the Laser Duo unit was activated for 180 s. The canals were then irrigated with 1.5 mL of distilled water. Before laser use, the output power was checked using the Laser Check (MM Optics, São Carlos, Brazil) power meter (Figure 1.8).

**Table 1:** Additional disinfection protocols according to experimental group

	PUI (n=10)	EC (n=10)	XPF (n=10)	aPDT (n=10)	CI (n=10)
Instrument	E1 Irrisonic (20*	Easy Clean (30.04**	XP-endo Finisher (25***	Optical fiber (20^#^	NaviTip (30^##^
Activation	Ultrasound: Piezon 150^###^, 30 Hz	VDW Silver^∆^ motor, Reciproc mode	VDW Silver^∆^ motor, DR’s choice (100 gcm, 800 rpm)	DUO^#^ laser, 660 nm, 100 mW	Manual, 5 mL Luer-lock^##^ syringe
Position	1 mm short of WL	WL	WL	WL	2 mm short of WL
Movement	back and forth	back and forth	back and forth	back and forth	back and forth
Time (s)	60 (3 x 20)	60 (3 x 20)	60 (3 x 20)	180	60
Irrigation	6 mL of 2.5% NaOCl (2 mL before each activation) + 5 mL 17% EDTA for 5 min + 3 mL distilled water	6 mL of 2.5% NaOCl (2 mL before each activation) + 5 mL 17% EDTA for 5 min + 3 mL distilled water	6 mL of 2.5% NaOCl (2 mL before each activation) + 5 mL 17% EDTA for 5 min + 3 mL distilled water	6 mL of 2.5% NaOCl (2 mL before each activation) + 5 mL 17% EDTA for 5 min + 3 mL distilled water (1.5 mL before aPDT and 1.5 mL after)	6 mL of 2.5% NaOCl (2 mL before each activation) + 5 mL 17% EDTA for 5 min + 3 mL distilled water

*Helse Dental Technology, São Paulo, Brazil; **Easy, Belo Horizonte, Brazil; ***FKG, La Chaux-de-Fonds, Switzerland; ^#^MM Optics, São Carlos, Brazil; ^##^Ultradent, South Jordan, UT; ^###^Electron Medical Systems, Nyon, Switzerland; ^∆^VDW, Munich, Germany

#### Evaluation using CLSM

Immediately after disinfection, the two halves of the roots were separated and analyzed under CLSM. Five minutes before being taken to the Olympus Fluoview 1000 (Olympus Corporation, Tokyo, Japan) microscope, the samples were stained with 10 µL of a 1:1 SYTO 9 and propidium iodide solution (L-13152 Live/Dead Baclight Bacterial Viability kit; Life Technologies, Carlsbad, CA). The half that showed the biofilm more clearly in each sample was chosen for analysis. Three image sets, one from each third of the canal, with a stack depth of 2 µm and at a resolution of 512 x 512 pixels, were captured at a 60x magnification using an oil immersion lens and two 473-nm and 559-nm wavelength lasers. After image acquisitions using the LUT tool of the Olympus Fluoview Ver.4.2b Viewer, the background, which corresponded to the stained dentin, was removed (Figure 1.9).

After that, cell biovolume was measured using the BioImage_L software (The MathWorks, Natick, MA). The surface and volume distribution tool were used to check the biovolume of viable (green) and nonviable (red) cells in each third of each sample after excluding the first stack, which corresponded to the canal surface, and standardizing depth for the next four stacks (8 µm). Noise reduction was adjusted to 0.01 to minimize background staining. The percentage of viable cell biovolume to total cell biovolume (viable + nonviable cells) was then calculated, and the result was used for comparisons (Figure 1.10).

The Kolmogorov-Smirnov test did not confirm data normality. Therefore, the percentage of viable cells along all the root canal and in each of its thirds was compared between groups using the Kruskal-Wallis test. The Friedman test was used to compare cell viability in the different canal thirds in each group. The Dunn post-hoc test was used. The significance level was set at 5%, and data were analyzed using the GraphPad Prisma 7.04 (GraphPad Software, San Diego, CA) software.

## Results

**Figure 2:**
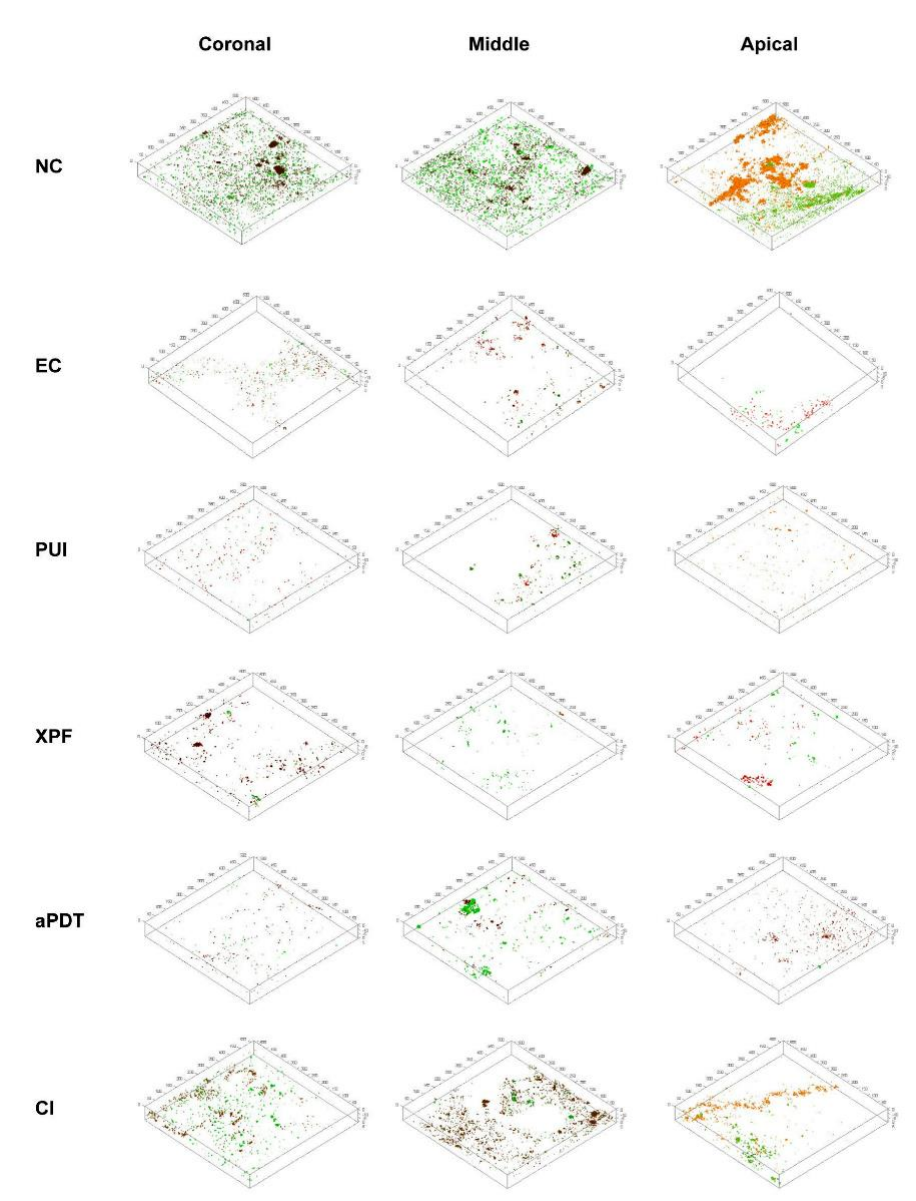
3-D reconstructions (biovolume) of biofilm population showing viable (green) and nonviable (red) cells according to third and experimental group.

The percentages of viable cell biovolume are summarized in Table 2, and viable and nonviable cell distribution is illustrated in Figure 2. In the same root canal third, there was no statistical difference in the percentage of the viable cell to total biovolume among the experimental groups (P>.05). EC, PUI, and aPDT samples had lower cell viability than control samples (P<.05). XPF and CI values were similar to those in the control group (P>.05). In the coronal third of samples in the EC, XPF, PUI, and aPDT groups, the percentage of viable cell biovolume was lower than in the control group (P<.05). In the middle and apical thirds, the percentage of viable cells was similar in all groups (P>.5) In the EC group, the percentage of viable cell biovolume in the coronal and middle thirds was lower than in the apical third (P<.05).

**Table 2:** Median percentage (%) (25th and 75th percentiles) of viable cell biovolume to total biovolume in experimental and control groups.

	EC	XPF	PUI	aPDT	CI	NC
Coronal	12.07^Aa^ (1.72-40.06)	18.05^Aa^ (1.08-59.31)	7.33^Aa^ (3.20-28.21)	20.45^Aa^ (4.58-42.85)	23.38^ABa^ (12.24-52.06)	61.30^Ba^ (43.14-70.44)
Middle	24.58^Aa^ (14.27-38.30)	46.49^Aa^ (10.70-91.86)	57.29^Aa^ (8.58-81.43)	20.33^Aa^ (9.30-88.11)	26.85^Aa^ (3.52-73.76)	63.48^Aa^ (50.83-74.83)
Apical	47.91^Ab^ (15.93-81.86)	49.59^Aa^ (31.18-74.56)	13.86^Aa^ (2.64-64.94)	50.01^Aa^ (8.78-85.93)	57.63^Aa^ (38.01-67.98)	47.09^Aa^ (28.29-75.74)
Total	29.99^A^ (7.32-47.64)	39.48^AB^ (5.06-62.82)	14.68^A^ (6.62-32.03)	27.96^A^ (9.36-59.02)	31.11^AB^ (23.77-60.07)	57.71^B^ (46.24-64.11)

*Different capital letters indicate significant difference in each row; different small letters indicate significant differences in each column (*P*≤.05).

## Discussion

This study provides information about the effect of different protocols to disinfect root canals on the cell viability of intraradicular biofilms formed *in situ*. The null hypothesis was rejected, as the biovolume of viable cells was smaller in the EC, PUI and aPDT groups than in the control group (P<.05). Although other studies also found a reduction in root canal infection when using supplementary disinfection protocols after canal cleaning and shaping^7^ ^,^ ^9^ ^,^ ^12^ ^,^ ^15^, to the best of our knowledge, this is the first report about the effect of complementary protocols on intraradicular multispecies biofilms formed *in situ*.

The experimental model used was selected to simulate clinical conditions. This study tested the effect of different protocols on multispecies biofilms formed *in situ*, which are more resistant to disinfection^21^. The negative control samples confirmed the presence of biofilm in the root canals after volunteers had worn the appliances, as demonstrated by other investigations about *in situ* biofilms^20^ ^,^ ^22^,. Monospecies biofilms, such as the one formed by *Enterococcus faecalis*, are commonly used to evaluate the antimicrobial effect of different protocols^19^ ^,^ ^23^ ^,^ ^27^. Multispecies biofilms can be produced *in vitro* by associating species such as *Enterococcus faecalis*, *Eikenella corroden*s, and *Streptococcus anginosus* ^17^ . However, these studies simplified the ecological conditions and did not reproduce the clinical scenario of endodontic infections^25^. Coaguila-Llerena et al.^18^ harvested subgingival biofilm to produce multispecies biofilm in vitro using the CDC reactor. The reactor allows for reproducible biofilms closely similar to those of endodontic infections. In the present research, microcosm biofilms induced and grown in situ allowed for analyzing the tested protocols' antimicrobial effect, embracing the individual heterogeneity of the samples. 

In this study, there were no differences between the disinfection protocols tested. However, biofilm cell viability in the EC, PUI, and aPDT groups was significantly different from that found in the control group. The samples in those groups had a lower percentage of viable cells than the untreated samples. Previous investigations also found a positive effect of EC in the reduction of root canal infection using different methods^19^ ^,^ ^13^. Our results revealed that, even in a multispecies biofilm formed *in situ*, the biovolume of viable cells was significantly reduced in comparison with that found in untreated samples when this automated instrument for irrigant agitation was used.

Studies found that PUI interferes significantly with the microbial component of the root canal system when compared with a control group^7^ ^,^ ^9^ ^,^ ^1017^ ^,^ ^24^. However, PUI’s efficacy in reducing microbial loads in samples collected from teeth with a primary infection seems to be greater than that found for EC^9^. Some methodological differences may explain these contradictory results. Unlike investigations that collected samples from the main root canal, this study estimated the proportion of viable cells inside dentinal tubules.

In agreement with our findings, other analyses using CFU counting *in vitro* multispecies biofilms found a reduction of microbial loads due to the use of aPDT^14^ ^,^ ^15^. An increase in the number of nonviable cells when aPDT is used, as found in a study using *E. faecalis* biofilm^8^, might be expected because of its deleterious effect on the cell membrane of microorganisms^16^. The comparison of aPDT samples with those in the control group seems to support this expectation, as the intervention reduced the percentage of viable cell biovolume.

The effect of XPF on the reduction of viable cell biovolume in the biofilm along all the root canals was not significant when compared with the control group. These results differ from those reported in studies that found a reduction in root canal microbial load after using XPF^7^ ^,^ ^11^ ^,^ ^12^. The differences may be explained by methodological differences, such as the characteristics of the biofilm under analysis. Differently from some studies^7^ ^,^ ^12^, we kept 2.5% NaOCl at 37º C before use to ensure that XPF worked perfectly while in the austenite phase. Other studies^10^ ^,^ ^12^ also increased NaOCl temperature to simulate clinical conditions. Besides, Teves et al.^17^ reported that the agitation of 4% NaOCl with XPF promoted more significant removal of the biofilm structure when evaluated by SEM compared to the irrigation with 4% NaOCl. Future studies should associate CLSM and SEM to provide a more comprehensive assessment of the effectiveness of XPF on intracanal biofilms.

Some studies investigated CI with sodium hypochlorite applied with a syringe and needle and found a positive antimicrobial activity and dissolution of organic matter, both pulp tissue and biofilm^26^ ^,^ ^27^. These results, however, were associated with several factors, such as needle characteristics, the reach of irrigation inside the canal, irrigant volume, and the anatomic complexity of the root canals^28^ ^,^ ^29^. In our study, the comparison with the control samples revealed that CI did not reduce the biovolume of viable cells, and its effect was similar to that of XPF. CI also had no effect on bacterial load in other investigations ^7^ ^,^ ^9^ ^,^ ^10^ ^,^ ^13^

CLSM was used to assess the effect of different protocols on the removal of multispecies biofilms formed *in situ* in root canals because it enables the counting of viable and nonviable bacteria in the three-dimensional structure of biofilm adhered to the dentin wall^21^ ^,^ ^30^. Results of this experimental model revealed that disinfection protocols for the root canal system promote different levels of ecological effects on multispecies biofilms formed *in situ*. However, it does not explain how large this effect has to be to reduce biofilm pathogenicity, which should be investigated in future studies.

In this study, EC, PUI, and aPDT protocols had significant ecological effects on intraradicular multispecies biofilms formed *in situ*, as they reduced the biovolume of viable bacterial cells when compared with untreated samples. Clinical studies should investigate the role of the reduction of viable bacterial loads in the root canal system in the success of endodontic treatment.
